# Spleen Stiffness as Predictor of Esophageal Varices in Cirrhosis of Different Etiologies

**DOI:** 10.1038/s41598-019-52407-y

**Published:** 2019-11-07

**Authors:** Carmen Fierbinteanu-Braticevici, Laura Tribus, Razvan Peagu, Ana Petrisor, Cristian Baicus, Dragos Cretoiu, Artur Pasternak, Gabriela Oprea, Adina Purcareanu, Alexandru C. Moldoveanu

**Affiliations:** 1 Medical Clinic II and Gastroenterology, Carol Davila University of Medicine and Pharmacy, Bucharest, 050474 Romania; 2 Department of Gastroenterology, Bucharest Emergency University Hospital, Bucharest, 050098 Romania; 3 Department of Clinical Epidemiology and Biostatistics, Carol Davila University of Medicine and Pharmacy Bucharest, Bucharest, 050474 Romania; 4 Department of Cellular and Molecular Biology and Histology, Carol Davila University of Medicine and Pharmacy, Bucharest, 050474 Romania; 5 Fetal Medicine Excellence Research Center, Alessandrescu-Rusescu National Institute for Mother and Child Health, Bucharest, 011062 Romania; 6 Department of Anatomy, Jagiellonian University Medical College, Krakow, 31-008 Poland; 7 Department of Internal Medicine, Bucharest Emergency University Hospital, Bucharest, 050098 Romania

**Keywords:** Liver cirrhosis, Liver cirrhosis

## Abstract

The purpose of this study is to determine whether esophageal varices (EV) can be identified through the evaluation of spleen stiffness (SSM) via acoustic radiation force impulse (ARFI). A total of 135 patients suffering from cirrhosis underwent a clinical exam, laboratory tests, abdominal ultrasound, liver stiffness (LSM) measurement, SSM evaluation and upper gastrointestinal endoscopy. Based on the endoscopy results, the patients were classified into three groups: those with no evident EV, those with small EV and those with varices needing treatment (VNT). Patients with EV of any grade had significantly higher average SSM values over those with no EV (3.37 m/s versus 2.79 m/s, p-value < 0.001), while patients with VNT showed an even greater difference (3.96 m/s versus 2.93 m/s, p-value < 0.001). SSM proved to be an excellent method of predicting patients with VNT.

## Introduction

Currently, patients suffering from cirrhosis are recommended upper gastrointestinal endoscopy (UGE) screening, in order to detect esophageal varices (EV). Unfortunately, UGE is an expensive, time-consuming method that is not well tolerated by patients. Consequently, various non-invasive methods of diagnosing EV have recently been proposed, the most promising one being the measuring of spleen stiffness (SSM). Our study proved that SSM, measured through acoustic radiation force impulse elastography, can consistently predict large EVs in cirrhotic patients, including those with a high risk of bleeding. Those with a higher bleeding risk are currently categorized as varices needing treatment (VNT) in the expanded Baveno VI guidelines. Our study proposes SSM as an alternative method for ruling out patients with VNT. This avoids the need for upper GI endoscopy, thus improving upon the Baveno VI criteria.

Portal hypertension (PH) as well as esophageal varices are major aggravations of hepatic cirrhosis, which are associated with increased mortality rates^1,2^. The gold standard method for measuring PH is the hepatic pressure venous gradient (HPVG), whilst the diagnosis of EV requires upper endoscopy^3,4^. Both of these investigations are invasive, not always well tolerated by patients, expensive, difficult to repeat and HPVG is also not widely available. Therefore, the need for noninvasive tools that accurately predict the presence and severity of PH and EV is crucial to help perform a proper prophylaxis of variceal bleeding^5–7^.

PH leads to splenic congestion, which induces architectural changes in the splenic arteries and veins, resulting in fibrosis and an increase in spleen stiffness (SSM)^5,8^. Recently, noninvasive techniques that measure SSM, in order to identify EVs and their hemorrhage risk are gaining more and more interest^5,7,9–12^. SSM can be evaluated through elastography techniques, such as shear wave elastography (SWE)^13^, transient elastography (TE)^14^, strain elastography and acoustic radiation force impulse (ARFI)^15^. These techniques are accurate in predicting the existence of EVs in most cases of cirrhosis with portal hypertension, including both viral and alcoholic cirrhosis^16–19^. TE is a useful method to detect high risk EVs, although it is limited by factors such as obesity and the ascites^5,20^. SWE is another good diagnostic method for those with clinically significant PH^21,22^.

Nevertheless, one of the most studied elastography techniques is ARFI, a new alternative investigation that evaluates tissue elasticity and that is reliable even in cases associating obesity or ascites^5,6^. ARFI is categorized as a displacement imaging technique, which uses a deep focused radiation force, induced by ultrasound. This measures the stiffness of a tissue, both quantitatively and qualitatively. The displacement is measured by comparing the locations of tissue echoes emitted before and immediately after the impulse^15^. Our study aimed to assess the clinical use of splenic stiffness (SSM), evaluated through ARFI, in patients suffering from cirrhosis. This was conducted in order to predict the existence and gravity of esophageal varices.

## Materials and Methods

### Study design

Our prospective study was performed on a series of patients with compensated cirrhosis from the Gastroenterology Department at the Bucharest Emergency University Hospital, between October 2016 and September 2017. The exclusion criteria included: decompensated cirrhosis (as evaluated by the presence of ascites, hepatic encephalopathy and the Child-Pugh Score), current treatment with beta blockers and previous or current variceal bleeding. The diagnosis of cirrhosis was established based on a combination of results from the clinical exam, laboratory blood tests, abdominal ultrasound and upper gastrointestinal endoscopy. Either positive histology from hepatic biopsy or LSM > 10 kPa on TE were required as inclusion criteria.

A study protocol was respected for each patient, which included a clinical exam, laboratory tests (hemogram, hepatic panel, serum albumin), abdominal ultrasound (highlighting the spleen diameter, portal vein diameter as well as the existence of ascites), liver and spleen ARFI elastography and upper gastrointestinal endoscopy. All tests were conducted within a maximum of 72 hours from each other. All methods were performed in accordance with the relevant institutional and national guidelines and regulations^23,24^. The study was approved by the Local Ethics Committee and informed consent forms were signed by each patient.

### Elastography measurement

Acoustic Radiation Force Imaging (ARFI) elastography was performed on each patient, after at least 12 hours of fasting, using the Siemens Acuson S2000 (Siemens AG) device and a dual B-mode ultrasound and quantitative elastography convex probe (Siemens 4C1). In order to conduct the measurements each patient was asked to hold his or her breath for 5 seconds. We carried out two separate sets of measurements – LSM and SSM respectively. Measurement sites were chosen 2 cm beneath the capsule of each organ. Each set of measurements was repeated at least ten times and the average value was chosen.

### Esophageal varices classification

Esophageal varices were evaluated for each patient, using upper gastrointestinal endoscopy and were classified into three groups, according to the expanded Baveno VI criteria^25^. Group 1 included patients with no esophageal varices, Group 2 – patients with low risk esophageal varices (varices that had a thickness of less than 5 mm) and Group 3 – patients with varices needing treatment (VNT – either large esophageal varices that had a thickness of more than 5 mm or varices displaying any signs of a high risk of bleeding: red wales, cherry red spots).

### Statistical analysis

**Figure 1:**
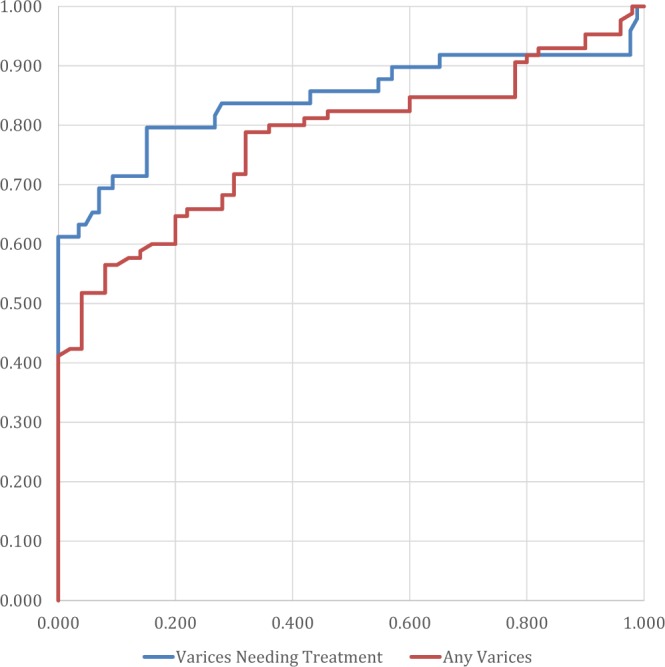
ROC curves for spleen elastography.

The results were inputted into a table using Microsoft Excel (Microsoft Corporation) and analyzed using Microsoft Excel, SPSS Version 23 (IBM Corporation) and MedCalc 19. The normality of the variables was assessed using histograms and the Shapiro-Wilk test. Univariate analysis for parameters with normal distribution was assessed using Independent Samples T Test, while for the parameters without normal distribution the Independent Samples Mann-Whitney U Test (for two categories) and Independent Samples Kruskal-Wallis Test (for more than two categories) were used. Multivariate analysis was performed using binary logistic regression. For each parameter, we assessed not only sensitivity and specificity, but also positive and negative predictive values, and positive and negative likelihood ratios at three distinct cut-off points – a cut-off based on the highest Youden’s J statistic point, a rule out cut-off (best negative likelihood ratio) and a rule in cut-off (best positive likelihood ratio). Cut-off values were selected based on the ROC curve and overall accuracy was determined using the area under the curve (AUROC). The strength of associations was calculated using the Spearman Rho test and multivariate analysis was performed on all parameters associated in univariate analysis. A p-value of <0.05 was considered statistically significant for all tests. The area under the ROC curve was interpreted as insignificant for results between 0.500–0.600, a poor predictor for results 0.600–0.700, a fair predictor for results 0.700–0.800, a good predictor for results 0.800–0.900 and an excellent predictor for results >0.900. The comparison between ROC curves was performed using the DeLong test, as illustrated in Fig. 1.

## Results

### Characteristics of patients

The study included a total number of 135 patients, out of which 77 were male and 58 were female. The patients were evenly distributed by age and gender, with no statistical difference between the groups. The etiology of cirrhosis was alcoholic (33 patients), hepatitis B (20 patients), hepatitis C (76), cryptogenic (5 patients) and primary biliary cirrhosis (1 patient). All enrolled patients were included in the study. Bivariate analysis identified several factors that were associated with the size of varices, including aspartate aminotransferase (p = 0.001), platelet count (p < 0.001), spleen diameter (p < 0.001), portal vein diameter (p < 0.001), liver elastography (p < 0.001) and spleen elastography (p < 0.001), as listed in Table 1.

**Table 1:** Patient characteristics and univariate analysis.

	No Varices (n = 50, 44%)	Small Varices (n = 36, 31%)	Varices Needing Treatment (n = 29, 25%)	p-value
Age	60.16 ± 10.16	57.61 ± 7.14	62.89 ± 9.82	0.091
Gender	23 F 27 M	14 F 22 M	14 F 15 M	0.733
Aspartate Aminotransferase	51.36 ± 38.21	48.44 ± 24.30	69.79 ± 26.95	0.001
Alanine Aminotransferase	44.66 ± 21.86	53.5 ± 28.81	56.51 ± 32.25	0.347
Platelet Count	188082 ± 70432	155958 ± 88680	102175 ± 47657	< 0.001
Spleen Diameter	110.96 ± 19.67	126.69 ± 22.58	141.69 ± 22.74	< 0.001
Portal Vein Diameter	10.16 ± 1.96	10.49 ± 1.68	11.69 ± 1.84	< 0.001
Hemoglobin	13.16 ± 1.25	13.28 ± 1.17	12.33 ± 1.79	0.210
Liver Elastography	2.00 ± 0.51	2.19 ± 0.54	2.55 ± 0.57	< 0.001
Spleen Elastography	**2.79 ± 0.41**	**3.02 ± 0.40**	**3.96 ± 0.37**	**< 0.001**

### Diagnostic performance of spleen stiffness

There is a stepwise increase in spleen stiffness values correlated with an increased severity of portal hypertension. Patients with esophageal varices of any grade had significantly higher average spleen elastography values than those with no varices (3.37 m/s versus 2.79 m/s, p-value < 0.001), while those with varices needing treatment had an even greater difference versus patients with no or small varices (3.96 m/s versus 2.93 m/s, p-value < 0.001). AUROC values were fair for the detection of any degree of esophageal varices (AUROC = 0.776, 95% CI = 0.698–0.853, p < 0.001) and excellent at distinguishing patients with varices needing treatment (AUROC = 0. 972, 95% CI = 0. 944–1.000, p < 0.001). The results of the liver elastography were poor for the detection of any degree of esophageal varices (AUROC = 0.682, 95% CI = 0.586–0.777, p < 0.001) and fair for distinguishing patients with varices needing treatment (AUROC = 0. 712, 95% CI = 0. 613–0.812, p < 0.001). The results of the splenic elastography were superior for varices needing treatment (p < 0.0001), but were not statistically significant for the diagnosis of any varices (p = 0.0768). The accuracy of spleen elastography for each test is detailed further in Table 2. The Spearman correlation test showed a stronger association for spleen elastography (r = 0.461, p < 0.001 for any grades of varices and r = 0.671, p < 0.001 for varices needing treatment) than any other investigated parameters, including liver elastography (which was second best, with a more modest r = 0.304, p < 0.001 for any grades of varices and r = 0.302, p < 0.001 for varices needing treatment). In the case of varices needing treatment, the variation of accuracy depending on prevalence is illustrated in Table 3. The multivariate analysis showed that spleen elastography was the sole parameter that was precisely correlated not only with the signs of an elevated risk of bleeding, but also with the existence of varices, irrespective of grade (p-values are presented in Table 4). The various etiologies of cirrhosis did not influence the spleen elastography results in our study (Table 5).

**Table 2:** The accuracy of spleen elastography.

	Any Esophageal Varices	Varices Needing Treatment (VNT)
AUROC (95% CI)	0.776 (0.698–0.853)	0.972 (0.944–1.000)
Balanced Cut-off	3 m/s: 79% Sensitivity; 67% Specificity; 80% PPV; 65% NPV; 2.39 LR + ; 0.31 LR-.	3.5 m/s: 93% Sensitivity; 86% Specificity; 64% PPV; 98% NPV; 6.64 LR + ; 0.08 LR-.
Rule In Cut-off	3.5 m/s: 47% Sensitivity; 96% Specificity; 80% PPV; 65% NPV; 11.75 LR + ; 0.55 LR-.	3.8 m/s: 55% Sensitivity; 98% Specificity; 89% PPV; 89% NPV; 27.5 LR + ; 0.46 LR-.
Rule Out Cut-off	2.5 m/s: 92% Sensitivity; 22% Specificity; 66% PPV; 61% NPV; 1.18 LR + ; 0.36 LR-.	3.2 m/s: 97% Sensitivity; 69% Specificity; 46% PPV; 99% NPV; 3.13 LR + ; 0.04 LR-.
Average Values	**3.37 m/s**	**3.96 m/s**

**Table 3:** Variation of accuracy for VNT based on different simulated prevalence.

Prevalence	Sensitivity	Specificity	PPV	NPV
50.0%	93.1%	0.0%	73.0%	0.0%
40.0%	93.1%	0.0%	64.3%	0.0%
30.0%	93.1%	31.8%	64.3%	77.8%
25.0%	93.1%	44.4%	64.3%	85.7%
20.0%	93.1%	57.1%	64.3%	90.9%
15.0%	93.1%	68.8%	64.3%	94.3%
10.0%	93.1%	79.7%	64.3%	96.7%

**Table 4:** The multivariate analysis results.

	Any Esophageal Varices		Varices Needing Treatment (VNT)	
	OR [95% CI]	p	OR [95% CI]	p
Aspartate Aminotransferase	0.998 [0.973–1.004]	0.134	0.997 [0.971–1.025]	0.851
Alanine Aminotransferase	1.015 [0.992–1.040]	0.209	1.008 [0.974–1.043]	0.651
Platelet Count	1.000 [1.000–1.000]	0.133	1.000 [1.000–1.000]	0.574
Spleen Diameter	1.033 [1.008–1.057]	0.009	1.021 [0.979–1.065]	0.337
Portal Vein Diameter	1.053 [0.813–1.363]	0.697	0.972 [0.548–1.722]	0.922
Hemoglobin	0.884 [0.633–1.234]	0.468	0.789 [0.511–1.218]	0.284
Spleen Elastography	3.976 [1.531–10.320]	0.005	1360.730 [34.183–54166.263]	0.000
Liver Elastography	1.468 [0.621–3.473]	0.382	0.955 [0.258–3.538]	0.945
Constant	0.01	0.038	0	0.003
	**AUROC = 0.845 [0.778–0.911]; Goodness of fit = 38.009, p < 0.001**		**AUROC = 0.974 [0.950–0.997]; Goodness of fit = 75.708, p < 0.001**

**Table 5:** Assessment of differences between different etiologies of cirrhosis.

	Any Esophageal Varices	Varices Needing Treatment (VNT)	Overall
Alcoholic	3.47 m/s	4.04 m/s	3.31 m/s
Hepatitis B	3.05 m/s	3.67 m/s	2.97 m/s
Hepatitis C	3.40 m/s	3.94 m/s	3.14 m/s
Other	3.63 m/s	4.15 m/s	3.05 m/s
Test	Independent Samples Kruskal-Wallis	Independent Samples Kruskal-Wallis	Independent Samples Kruskal-Wallis
p-value	0.078	0.383	0.301

## Discussions

One of the major complications of cirrhosis, regardless of its etiology is portal hypertension, a frequent cause of death for cirrhotic patients. Clinically significant portal hypertension (CSPH, hepatic venous pressure gradient ≥ 10 mmHg) is the main cause of cirrhosis decompensation and it can influence the survival of cirrhotic patients. The complications of CSPH include ascites, hepatic encephalopathy and bleeding from gastroesophageal varices, which is still associated with a high mortality rate of 10–20%^4^ at six weeks. Therefore, the assessment of portal pressure is a constant concern that influences the outcomes in both stages of cirrhosis: compensated or decompensated. HVPG and upper endoscopy are the gold-standard techniques for the diagnosis of portal hypertension and esophageal varices. However, their invasiveness makes them rarely accepted by patients. The non-invasive tests are able to provide similar information and are more welcomed. Previous studies, that have used HVPG as a reference, have shown a significant association between the LSM values and the risk of portal hypertension development in cirrhotic patients, by assessing the liver stiffness with the help of transient elastography^26–32^. Also, there are several studies that analyzed the predictive value of TE for esophageal varices and concluded that there is a significant correlation between TE values and the existence of esophageal varices^33–37^. However, in severe cirrhosis, the link between LSM and HVPG lessens as a result of cumulating factors, such as mechanical changes in the liver, mainly fibrosis, and the dynamic feature of PH (intrahepatic vasoconstriction and splanchnic vasodilation) that cannot be evaluated by TE^38^. A recent meta-analysis, that evaluated the performance of TE for detecting esophageal varices in cirrhotic patients, concluded that TE is not suitable for implementation in the clinical practice due to varying cut-off values and different etiologies^39^. The morphological changes of the spleen in portal hypertension, such as the vascular congestion angiogenesis, the lymphoid hyperplasia and the fibrosis have justified the use of spleen stiffness as another surrogate parameter of PH in cirrhosis^40,41^.

As a result, our study wanted to determine the predictive value of SSM measurement through virtual touch quantification, in the assessment of the existence and gravity of esophageal varices (EV) in patients suffering from cirrhosis of different etiologies. Upper digestive endoscopy was used as a comparison for the efficiency of this method. Spleen stiffness measurements were successfully carried out in all enrolled patients, resulting in a wide range of values that varied between 2.18 m/s and 4.77 m/s. The results showed a stepwise increase in splenic stiffness with the increasing size of the varices (p < 0.001). The accuracy of SSM in predicting the presence of EV was demonstrated to be fair - good, with an AUROC of 0.776 and a good sensitivity and specificity (Se 72%, Sp 71%). The values for confirming or excluding the presence of the varices were: ≥ 3.50 m/s and < 2.50 m/s respectively. There are many studies that deemed the importance of spleen stiffness as being equivalent to that of the endoscopy in predicting esophageal varices^5,17,42^. The majority of these used TE when performing SSM (TE-SSM)^43–47^.

There are at least two limitations for TE-SSM: the high percentage of failed measurements and the variability of the values with respect to the etiology of the liver diseases. In addition, there is one more limitation regarding the upper limit of the cut-off value of 75 kPa, considering that SSM frequently exceeds this value^5,42,45,46^.

There are several studies that performed ARFI for the assessment of spleen stiffness, alone^10,18,48–50^ or in comparison with TE^5^. Only two of those took HVPG as a reference for evaluating the spleen stiffness measurement performance^51,52^. Both of them revealed the remarkable accuracy of SSM in predicting clinically relevant portal pressure values. Most of the studies were aimed at evaluating the connection between SSM, measured through ARFI, and the presence and stage of the esophageal varices. As demonstrated in our study, there is a considerable correlation between ARFI-SSM and the existence of EVs in patients suffering from cirrhosis. Also, our study showed no significant differences between the optimal cut-off values of ARFI-SSM in predicting the existence of varices: 3.10 m/s^48^ 3.18 m/s^51^/ 3.16 m/s^5^ and 3.02 m/s in our study. With regard to the performance of ARFI-SSM in predicting the severity of PH, expressed as the size of varices and the risk of bleeding, our results showed an excellent performance, AUROC = 0.972, in recognizing patients with varices needing treatment. The cut-off values of ARFI-SSM to rule out and rule in the varices at risk for bleeding were < 3.20 and ≥ 3.80 ms/s respectively. For a cut-off value of 3.20 m/s the predictive negative value is remarkable (99%) for excluding the cases with varices needing treatment. Another advantage of ARFI, underlined by all these studies, is the high feasibility of this method, without measurement failures like in the case of TE-SSM. However, there is one study that did not find ARFI-SSM to be a good parameter in evaluating the existence and gravity of EV^53^. On the other hand, the same group developed a predictive score that analyzed LSM and SSM, using ARFI measurements, in cirrhotic patients associating ascites, with a good accuracy in anticipating significant EV^50^.

One concern was raised regarding SSM as a complementary method in evaluating the existence of EVs in patients suffering from cirrhosis: the loss of correlation in alcohol induced cirrhosis^49^. Without a clear reason for this result, the authors have considered the smaller dimension of the spleen that characterized alcoholic cirrhosis to be a probable cause, as compared to viral or non-alcoholic steatohepatitis-cirrhosis. Our study did not confirm this hypothesis, because it found no differences between the diverse etiology of cirrhosis, including the one induced by alcohol (p = 0.301).

Another purpose of our study was to analyze the other parameters that were linked to the existence and severity of EV, in patients suffering from cirrhosis. In this regard, bivariate analysis showed a significant correlation, alongside SSM, of the platelet number, splenic size, portal vein diameter, AST values and ARFI - liver elasticity (p < 0.001). All these parameters were related not only to the presence and size of the varices, but also to the varices needing treatment. However, after multivariate analysis, SSM remained the only parameter that was highly associated with the presence and size of EVs and also with their risk of bleeding. There was another independent variable associated only with one, but not with all characteristics of esophageal varices: the splenic diameter used for detecting the presence of EV (p = 0.009). However, SSM was indisputably the strongest predictor for all endoscopic aspects of EV, from their presence, to the detection of varices needing treatment. Other studies also found ARFI-SSM to be a unique independent parameter for EV prediction, according to multivariate analysis^48,49^.

One limitation of our study is the uneven distribution of cirrhosis etiology, having only a few cases with NASH-cirrhosis, autoimmune and biliary cirrhosis. Therefore, it is difficult to assess the efficacy of splenic stiffness in different etiologies of cirrhosis. Another limitation is the lack of a standard protocol for performing the measurements for spleen stiffness. Usually, the measurements were made 2 cm under the splenic capsule, mostly in the middle part of the spleen. In the study of Rizo *et al*.^41^ the measurements were extensively performed throughout the entire splenic parenchyma, in order to be more representative.

However, we consider that ARFI spleen stiffness measurement is an optimal method to use in the clinical practice, for the screening of cirrhotic patients, for esophageal varices. By easily and reliably diagnosing high-risk varices, ARFI can discriminate variceal bleeders from non-bleeders. We believe that our study may contribute to recognizing splenic stiffness as a novel parameter and an unlimited tool for gastroenterologists to use in the screening of the cirrhotic population, reducing the number of endoscopic examinations and, as a result, improving the Baveno VI criteria.
